# A semi-supervised approach for the integration of multi-omics data based on transformer multi-head self-attention mechanism and graph convolutional networks

**DOI:** 10.1186/s12864-024-09985-7

**Published:** 2024-01-22

**Authors:** Jiahui Wang, Nanqing Liao, Xiaofei Du, Qingfeng Chen, Bizhong Wei

**Affiliations:** 1 School of Computer and Information Security, Guilin University of Electronic Technology, No. 1 Jinji Road, Guilin City, 541004 Guangxi Zhuang Autonomous Region China; 2 School of Computer, Electronics and Information, Guangxi University, No. 100 East University Road, Nanning, 530004 Guangxi China; 3 School of Medical, Guangxi University, No. 100 East University Road, Nanning, 530004 Guangxi China

**Keywords:** Multi-omics, Semi-supervised learning, Multi-head self-attention mechanism, Graph Convolutional Networks

## Abstract

**Background and objectives:**

Comprehensive analysis of multi-omics data is crucial for accurately formulating effective treatment plans for complex diseases. Supervised ensemble methods have gained popularity in recent years for multi-omics data analysis. However, existing research based on supervised learning algorithms often fails to fully harness the information from unlabeled nodes and overlooks the latent features within and among different omics, as well as the various associations among features. Here, we present a novel multi-omics integrative method MOSEGCN, based on the Transformer multi-head self-attention mechanism and Graph Convolutional Networks(GCN), with the aim of enhancing the accuracy of complex disease classification. MOSEGCN first employs the Transformer multi-head self-attention mechanism and Similarity Network Fusion (SNF) to separately learn the inherent correlations of latent features within and among different omics, constructing a comprehensive view of diseases. Subsequently, it feeds the learned crucial information into a self-ensembling Graph Convolutional Network (SEGCN) built upon semi-supervised learning methods for training and testing, facilitating a better analysis and utilization of information from multi-omics data to achieve precise classification of disease subtypes.

**Results:**

The experimental results show that MOSEGCN outperforms several state-of-the-art multi-omics integrative analysis approaches on three types of omics data: mRNA expression data, microRNA expression data, and DNA methylation data, with accuracy rates of 83.0% for Alzheimer's disease and 86.7% for breast cancer subtyping. Furthermore, MOSEGCN exhibits strong generalizability on the GBM dataset, enabling the identification of important biomarkers for related diseases.

**Conclusion:**

MOSEGCN explores the significant relationship information among different omics and within each omics' latent features, effectively leveraging labeled and unlabeled information to further enhance the accuracy of complex disease classification. It also provides a promising approach for identifying reliable biomarkers, paving the way for personalized medicine.

## Introduction

The advent of cutting-edge sequencing technologies has facilitated the rapid acquisition of voluminous data from various omics domains, including mRNA expression, DNA methylation, and microRNA expression data. The utilization of diverse omics data enables the multifaceted representation of the biological processes underpinning complex diseases. In the early stages, the majority of researchers primarily employed traditional machine learning methods for the "unidimensional" analysis of single omics data in the study of disease mechanisms [1]. mRNA gene expression was the most prevalent focus [2–4]. However, for the intricacies of biological complexity, the analysis of single omics data remains inherently limited [5]. Current research has shown that, in comparison to experiments conducted using single omics data, the utilization of multi-omics data sources permits a more comprehensive analysis of disease risk, prognosis, and enhances predictive capabilities [6–9]. The integration analysis of multi-omics data supplements the information from various omics domains, compensating for the limitations of singular omics datasets and providing a more comprehensive research perspective for disease classification [10].

Some of the existing multi-omics studies have been rooted in unsupervised learning approaches. Chen Meng et al. [11] proposed multiple co-inertia analysis (MCIA) method. This method employs a covariance optimization criterion to simultaneously project multiple datasets (such as genes and proteins) onto a common one-dimensional space. It transforms distinct sets of features to a uniform scale, facilitating the extraction of features relevant to sample clusters. Michael J et al. [12] introduced the Joint and Individual Variation Explained (JIVE) method as an exploratory dimensionality reduction tool. JIVE dissects multi-omics datasets and integrates them to acquire comprehensive information regarding breast cancer. However, in recent years, due to the rapid advancement in medical technology and the accumulation of relevant data, the volume of biological features and trait data exhibited by individuals has increased significantly. Utilizing unsupervised learning is no longer sufficient to meet the demands of integrated analysis for multi-omics data. Instead, supervised learning methods in multi-omics, which incorporate sample label information, are increasingly applied in disease prognosis and prediction research. ZI-YI YANG et al. [13] proposed the Multi-Modal Self-Paced Learning (MSPL) algorithm for the integration of multi-omics data. This approach employs a sparse logistic regression classifier in cancer subtype classification and identifies latent biological features. Xu et al. [14] employed a novel hierarchical integrated deep flexible neural forest framework (HI-DFNForest) to integrate three types of omics data: DNA methylation, gene expression, and microRNA expression data, successfully classifying ovarian subtypes. Yang et al. [15] introduced the Subtype-GAN method, a deep adversarial learning approach with multiple inputs and outputs, which utilizes consistency clustering and Gaussian mixture models to identify molecular subtypes of tumor samples. Singh et al. [16] proposed Data Integration Analysis for Biomarker Discovery (DIABLO), a multivariate dimensionality reduction method that maximally utilizes covariance and latent components information within linear combinations of features from multiple omics sources for prediction. While these methods have demonstrated effectiveness, they have not fully considered the relationships among different omics data types and have overlooked inter-patient correlations. Given the importance of leveraging both inter-patient correlations and inter-omics relationships, Wang T et al. [17] introduced a Multi-Omics Graph Convolutional Networks (MOGONET) algorithm. This algorithm employs cosine similarity to compute a patient correlation network as input for Graph Convolutional Networks (GCN) and explores cross-omics correlations in the label space using View Correlation Discovery Network (VCDN) after GCN output. Li et al. [18] proposed a multi-omics integration method based on graph convolutional networks (MOGCN). This method utilizes autoencoders for dimensionality reduction, integrates Copy Number Variations (CNV), mRNA, and Reverse Phase Protein Array (RPPA) data, and employs the results of Similarity Network Fusion (SNF) to construct a patient similarity network as GCN input.

In summary, while the aforementioned methods consider inter-patient correlations and inter-omics relationships and have, to a certain extent, improved the accuracy of complex disease classification, they still face certain challenges. Firstly, many data types have a limited number of labeled samples and a larger number of unlabeled samples. Traditional supervised learning methods do not directly leverage information from unlabeled nodes, and classic GCN methods do not utilize unlabeled node information directly during the training process [19], restricting information propagation and diminishing model generalization capabilities. Secondly, previous feature processing methods have not accounted for the unique subspaces of each omics data type and the multiple associations and dependencies among latent features within different omics data. This oversight may lead to results that are biased towards specific omics data types or particular features. Addressing these issues, we propose a novel ensemble learning model for analyzing multi-omics data. It fully exploits the correlations within the latent features of each omics data and inter-omics relationships, as well as the information from unlabeled nodes. The model is constructed by utilizing Transformer encoding modules to explore the potential advanced features and inherent relationships within each omics data and between different omics. Subsequently, it employs Similarity Network Fusion (SNF) to build a patient similarity fusion network. Finally, it employs Self-Ensembling Graph Convolutional Networks (SEGCN) for training, simultaneously utilizing labeled and unlabeled data to better capture the overall characteristics and underlying structures of the data, thereby enhancing model generalization capabilities. Additionally, this model can identify important omics features and biomarkers, offering interpretability and providing a research methodology for future clinical.

## Methods

**Fig. 1:**
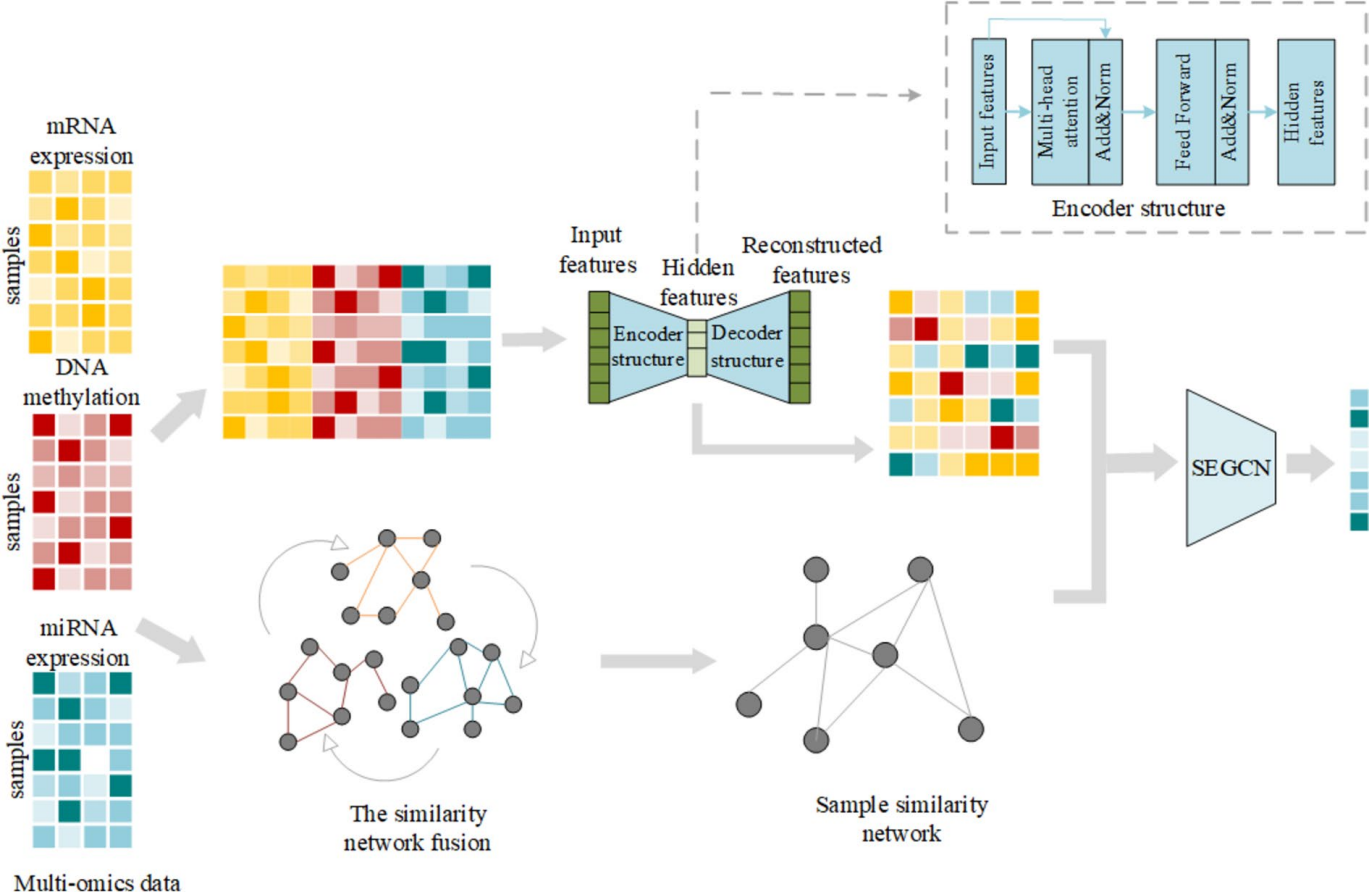
MOSEGCN Framework

In this section, we shall provide a comprehensive exposition of the content pertaining to the multi-omics data integration learning model, MOSEGCN. Figure 1 illustrates the framework of MOSEGCN, which primarily comprises three components: the Transformer encoding module tailored for multi-omics features learning, the module dedicated to constructing a patient-fusion similarity network, and the ultimate SEGCN classification module.

### Transformer

The Transformer model was initially employed in natural language processing [20]. Over time, it underwent adaptations for image recognition and object detection, demonstrating its efficacy [21–25]. The fundamental Transformer architecture comprises an input layer, multi-head self-attention blocks, normalization layers, feedforward layers, and residual connection layers. Essentially, it embodies an Encoder-Decoder framework [26]. Key components within the Transformer model are the multi-head self-attention mechanism and the autoencoder. The autoencoder is proficient at discerning latent features from input data, offering an effective approach to amalgamate distinct features [27]. The multi-head self-attention mechanism is an enhanced algorithm building upon common attention mechanisms. Its virtue lies in its ability to apprehend the intrinsic correlations among various features across different positions and data points [28]. This algorithm excels in capturing the inner relationships among diverse features and mitigating reliance on external information. It notably accentuates critical attributes for classifying related disease subtypes, with a particular emphasis on valuable insights from the test set, comprising unlabeled nodes.

Given that identical samples in the data encompass features from diverse omics, the experimental approach necessitates the full exploitation of concealed information within each omic, inter-omic latent feature information, and multifarious associations and dependencies among features. Consequently, this experimental method introduces a self-attention layer prior to the encoder's output layer. This layer comprehensively captures positional information from the input data, explores correlations between latent features within each omic and across different omics, and assesses the significance of features within each modality. The residual connections [29] facilitate the flow of information within the model, and normalization layers [30], positioned after the self-attention layer and before the feedforward network, enhance training stability and expedite convergence.

#### Autoencoder

The autoencoder is an unsupervised neural network model employing the backpropagation algorithm. Typically, it consists of two modules: the encoder and the decoder. The encoder maps input data into a lower-dimensional latent space, which is then mapped back to the original data space by the decoder [31]. Given that both the latent features learned within each omics data's exclusive subspace and the latent features across different omics contribute to the model [32], and considering that the multi-head self-attention mechanism accounts for correlations among positions in input data, the experimental setup utilizes feature data concatenated from three modalities of the original input $X\in {R}^{N{\text{xP}}}$, where N represents the number of samples, $P=\left[{{\text{p}}}_{1},{p}_{2}\cdots {p}_{i}\right]$ where ${{\text{p}}}_{{\text{i}}}$ represents the features possessed by the i-th modality. The entire process of the autoencoder can be represented as follows:and

1 $$Decoder\left(E{\text{ncoder}}\left(X,{\theta }_{e}\right),{\theta }_{{\text{d}}}\right)=\widetilde{X}$$

where $\widetilde{X}$ is the reconstruction representation with the same shape as X, ${\theta }_{e}$ and ${\theta }_{d}$ are the parameters of the encoder and decoder neural networks, respectively. $E{\text{ncoder}}\left(X,{\theta }_{e}\right)=H\in {R}^{N\times K}$ , H is referred to as the latent representation of X, meaning the encoder maps N samples from a P-dimensional space to a K-dimensional space. Finally, the autoencoder trains the encoder and decoder by minimizing the reconstruction error to learn useful representations of data both within the same modality and across different modalities: $$\begin{array}{c}{\text{argmin}}\\ {\theta }_{e},{\theta }_{d}\end{array}\Vert X-\widetilde{X}\Vert^2_{F}$$. In this experiment, only the encoder function block is used to obtain the ultimately valuable features.

#### The multi-head self-attention mechanism

The multi-head self-attention mechanism builds upon the foundation of the self-attention mechanism, introducing multiple attention heads to fully leverage input information in capturing various associations and dependencies within features. This enhances the model's comprehension of feature information [33]. Since the features extracted by the autoencoder may contain some redundancy or irrelevant elements, potentially overlooking hidden information, this experiment employs the multi-head self-attention mechanism to further learn the internal correlations among features at various positions. This, in turn, assigns higher weights to crucial features in the context of cancer subtype classification, aiding the neural network in feature selection [34]. In conclusion, the inclusion of the multi-head self-attention mechanism allows for the identification of pivotal features vital for predicting events based on critical information from different omics and individual omics data. The computational formula for multi-head attention is as follows:

2 $$MultiHead\left(Q,K,V\right)=Concat\left(hea{d}_{1},\cdots ,hea{d}_{h}\right){W}^{o}$$

3 $$\begin{array}{c}Hea{d}_{i}=soft {\text{max}}\left(\frac{{Q}_{\mathrm{i }}\times {K}_{i}^{T}}{\sqrt{{d}_{K}}}\right){V}_{i}\\ \begin{array}{c}{Q}_{i}=H\times {W}_{i}^{Q}\\ {K}_{i}=H\times {W}_{i}^{K}\end{array}\\ {V}_{i}=H\times {W}_{i}^{V}\end{array}$$

${W}^{o}$ represents the output transformation matrix, h denotes the number of heads, and ${{\text{head}}}_{{\text{i}}}$ signifies the output of the i-th head ${Q}_{i},{K}_{i}$and ${V}_{i}$ correspondingly emerge from the linear transformations of the latent vector H, with ${W}_{i}^{Q}\in {R}^{{d}_{H}\times {d}_{Q}},{W}_{i}^{K},\in {R}^{{d}_{H}\times {d}_{K}},{W}_{{i}}^{V}\in {R}^{{d}_{H}\times {d}_{{\text{V}}}}$ representing the parameter matrix.

### SNF

The Similarity Network Fusion (SNF) [35] method employs pairwise correlations between samples to construct sample similarity matrices for each omics data type. In this experiment, the neighborhood size is set to 30, and the hyperparameter σ is assigned a value of 0.5. Distinct sample similarity networks are constructed for different omics data types. Subsequently, leveraging the complementary information from different omics data types, the three distinct similarity networks obtained earlier are computed and fused, eliminating weak connections. Ultimately, a comprehensive view of the disease is established. In this final comprehensive view, nodes represent samples, and edges indicate pairwise similarities between samples. The experiment implements this module in the PYTHON software using the SNFpy package, facilitating graph integration analysis.

### Self-ensembling graph convolutional networks

To enhance model performance by fully leveraging the information from unlabeled nodes, our experiment employs the Self-Ensembling Graph Convolutional Networks (SEGCN) method [36]. SEGCN represents a potent and highly reliable self-ensembling learning mechanism that combines GCN (Graph Convolutional Networks) and Mean Teacher in a semi-supervised task. GCN, a deep learning model designed for processing graph-structured data, operates on the fundamental principle of defining convolutional operations using the graph's adjacency matrix. However, the classical GCN algorithm, functioning as a localized spectral graph convolution with first-order approximations, explores only half of the unannotated information [19]. Mean Teacher [37] comprises both a teacher model and a student model. The inconsistency between the student's outputs under slight perturbations and the teacher model's outputs serves as a robust clue for classifying cancer subtypes in unlabeled nodes. In other words, unlabeled nodes can provide highly effective gradients under the supervision of consistency loss to train the model. In this mutually reinforcing process, both labeled and unlabeled sample information is effectively propagated for gradient-based training of GCN. The GCN model [38] obtains the output of a single convolutional layer by configuring the adjacency matrix $A$ and $X$ input features, $\widetilde{A}=A+{I}_{N}$, ${\widetilde{D}}_{ii}=\sum_{j}{\widetilde{A}}_{ij}$, $\Theta$ represented as trainable model parameters:$Z={\widetilde{D}}^{{-}_{2}^{1}}\widetilde{A}{\widetilde{D}}^{{-}_{2}^{1}}X\Theta$.

SEGCN comprises both a student model $f\left(\Theta_{s} \right)$ and a teacher model$f\left({\Theta }_{t}\right)$,${\Theta }_{s}$, ${\Theta }_{t}$ each with their respective weights. Given labeled data ${D}_{L}={\left\{{x}_{i}^{L},{y}_{i}^{L}\right\}}_{i=1}^{{N}_{L}}$ and unlabeled data${D}_{U}={\left\{{x}_{i}^{u}\right\}}_{i=1}^{{N}_{U}}$. In this experiment, a normalized adjacency matrix A is constructed based on data relationships, x represents the labeled samples. $f{\left(A,x;{\Theta }_{s}\right)}_{c}$ represents the predicted probabilities of the student classifier for the c classes, while ${y}_{c}$ represents the ground truth probabilities for the c classes. In a noise-free environment, the cross-entropy loss for labeled data under supervision is expressed as:

4 $${\ell}_{CE\left(\Theta_{s} ,A,x,y\right)}=-\sum_{c=1}^{C}{y}_{c}logf{\left(A,x;{\Theta }_{s}\right)}_{c}$$

In this experiment, model perturbation $f{\prime}\left(.\right)$ is achieved by adding only one dropout layer with a dropout rate set to 0.5. The unsupervised consistency loss penalizes the discrepancies between the student's predicted probabilities $f{\prime}\left(A,x;{\Theta }_{s}\right)$ and those of the teacher $f\left(A,x;{\Theta }_{t}\right)$. The formulation of the unsupervised consistency loss is as follows:

5 $${\ell}_{cons}\left({\Theta }_{t},{\Theta }_{s},A,x\right)=\sum_{x\in {D}_{L}U{D}_{U}}\Vert f\left(A,x;{\Theta }_{t}\right),f{\prime}\left(A,x;{\Theta }_{s}\right)\Vert$$

The overall loss of SEGCN comprises both supervised and unsupervised losses, given as follows: $L\left({\Theta }_{t},{\Theta }_{s},A,x,y\right)=\sum_{\left(x,y\right)\in {D}_{L}}{\ell}_{CE}+\lambda \sum_{x\in {D}_{L}U{D}_{U}}{\ell}_{cons}$ Here, the parameter $\lambda >0$ controls the relative importance of the unsupervised loss in the overall loss. The weights of the teacher model are updated using the exponential moving average of the student's real-time weights, ${\Theta }_{t}^{s+1}=\alpha{\Theta }_{t}^{s}\left(1-\alpha\right){\Theta }_{s}^{s+1}$, with $a$ being the smoothing coefficient and s being the current step. $\alpha$ and $\lambda$ are set to their default values in SEGCN, with the number of GCN layers set to 2 to demonstrate that the model achieves its best performance with two layers [36].

## Results

In this section, the performance of the proposed MOSEGCN model is evaluated and compared with other state-of-the-art methods:1. Random Forest (RF): Constructing multiple decision trees and combining their predictions for final classification. 2. k-Nearest Neighbors Classifier (KNN): Classifying based on the labels of neighboring samples for the sample to be predicted. 3. L1 Regularized Linear Regression (Lasso): Considering relationships and differences between multiple categories simultaneously for multi-omics data fusion classification. 4. XGBoost: Implementing a classifier based on gradient-boosted decision trees. 5. MoGCN: Utilizing autoencoders (AE) to learn multi-omics features for GCN classification. 6. MOGONET: Jointly learning the specificity of omics and the correlation of cross-omics after pre-classification using GCN. 7. Combining Transformer encoding modules with GCN to create a novel model for cancer classification. 8. Semi-Supervised SVM (S3VM): This is an extended approach to Support Vector Machines (SVM) that enhances model performance by simultaneously leveraging labeled and unlabeled data. 9. SEGCN: A deep learning model designed for semi-supervised tasks, incorporating self-ensembling techniques to boost performance.

MOSEGCN is first compared with these nine methods on two benchmark cancer datasets. Subsequently, it is validated for applicability and effectiveness using a multi-omics dataset of glioblastoma multiforme, which contains four cancer subtypes and a total of 274 samples. Finally, the model's sensitivity analysis is employed to identify important biomarkers.

### Data preparation

We utilized preprocessed benchmark multi-omics cancer datasets, namely ROSMAP and BRCA [17], to assess the performance of our experimental model across different cancer classification tasks. In particular, the BRCA dataset encompasses classification of invasive breast cancer (BRCA) PAM50 subtypes, including normal, basal, human epidermal growth factor receptor 2 (HER2)-enriched, Luminal A subtype, and Luminal B subtype.

The multi-omics dataset for Glioblastoma Multiforme (GBM) was obtained from an open-access website accessed on May 16, 2023, at. This dataset comprises four files: three data groups (i. e., gene expression, DNA methylation expression, and microRNA expression), along with one clinical dataset. To effectively analyze multi-omics data, the following preprocessing steps were undertaken. First, samples common to all four data groups were selected, and features devoid of signals (zero mean) were further filtered. Second, the most significantly differentially expressed genes (the top 25% with the highest variance) were selected and MinMaxScaler-transformed for subsequent analysis. Regarding microRNA expression data, due to the limited number of microRNA and features available, no selection was performed. The clinical dataset retained labels for the four cancer subtypes of the samples. The experiment utilized a 7:3 split for training and testing, repeated 30 times, with average measurement results reported. Table 1 provides a concise overview of the three datasets.

**Table 1:** Dataset Overview

Dataset	Categories	Number of features for mRNA	Number of features for methylation	Number of Features for microRNA	Number of labeled nodes	Number of unlabeled nodes
BRCA	Normal-like:115, Basal-like: 131, HER2-enriched:46, LuminalA:436, Luminal B: 147	1000	1000	503	612	263
ROSMAP	NC:169, AD:182	200	200	200	245	106
GBM	Classical:71, Mesenchymal:47, Proneura:84, Neural:72	3613	1500	534	191	83

### Hyper-parameter setting

**Fig. 2:**
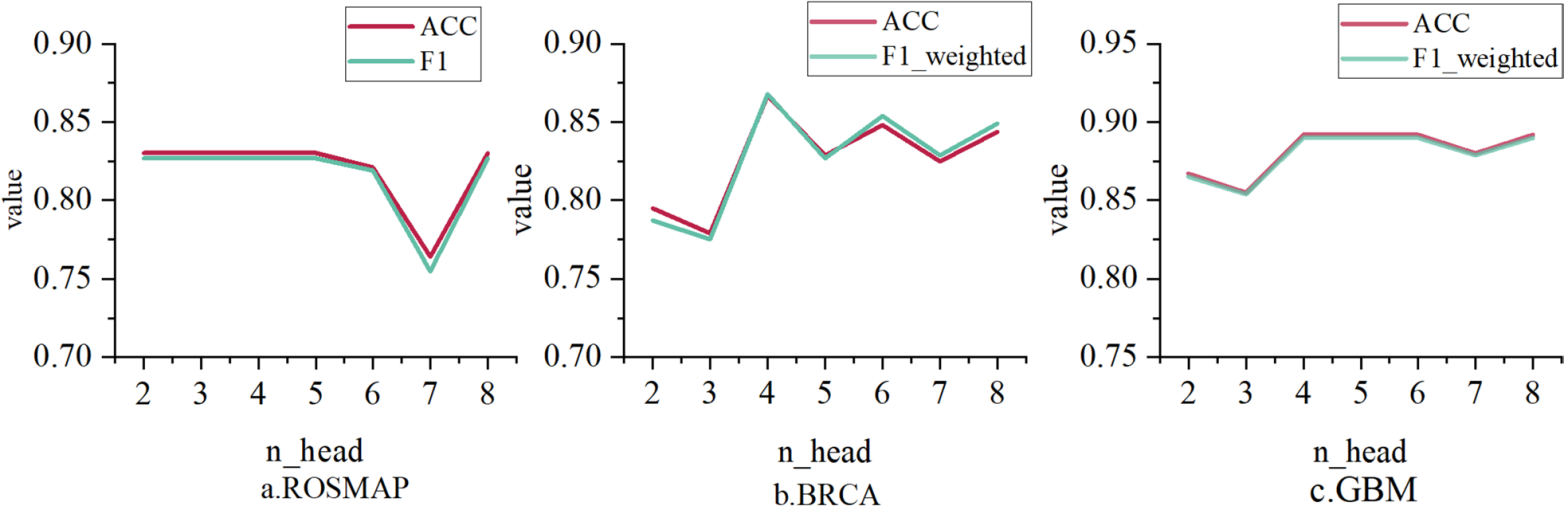
Evaluation Metrics as a Function of n _head Variation

The performance of MOSEGCN is directly influenced by the settings of hyperparameters, and one of these settings is the number of attention heads in the multi-head attention mechanism. Having a higher number of attention heads can potentially lead to increased computational complexity, training requirements, and memory consumption. Additionally, the interaction and integration of information between attention heads may become more intricate, making the model harder to optimize. Conversely, having a lower number of attention heads might limit the model's expressive power and feature extraction capabilities, preventing it from capturing complex relationships and patterns within multi-omics data. Selecting an appropriate number of attention heads requires striking a balance between the model's expressive capacity and computational complexity. Therefore, this study undertakes experimentation to fine-tune and determine the optimal number of attention heads. As depicted in Fig. 2, it becomes evident that when *n*_head = 4, the three datasets achieve the most outstanding classification performance within the model.

### Dataset analysis

(Tables 2, and 3) present the test set accuracy results for the two benchmark cancer datasets, ROSMAP and BRCA. In the binary classification task for ROSMAP, the experiment employs accuracy (ACC), F1 score (F1), area under the receiver operating characteristic curve (AUC), Precision and Recall as evaluation metrics. For other multi-class datasets, accuracy (ACC), weighted F1 score (F1_weighted), macro F1 score (F1_macro), Precision and Recall are utilized. The experimental findings demonstrate that MOSEGCN outperforms in all benchmark test datasets. The accuracy rates for ROSMAP and BRCA reach 83.0% and 86.7%, respectively. Compared to the latest MOGONET method, MOSEGCN shows an improvement of 3.0% and 6.1% in accuracy for these datasets, indicating outstanding classification performance in common complex diseases like breast cancer and Alzheimer's disease. MOSEGCN consists of two crucial components: the Transformer encoding module, which learns high-level features and their inherent correlations within and between different omics data types, and SEGCN, which employs labeled and unlabeled information for final classification. To validate the necessity of each component, this experiment combines the Transformer encoding module with GCN for classification purposes. The results in Tables 2, and 3 demonstrate that the combination of the Transformer encoding module and GCN outperforms the integrated model MOGCN [18], which utilizes AE and GCN modules, particularly in handling multiple omics data sets. Similarly, the evaluation metrics of the MOSEGCN model, incorporating the Transformer encoding module, surpass those of the semi-supervised model SEGCN. This underscores the effectiveness of the Transformer encoding module in integrating multiple omics data sets, showcasing its enhanced capability to capture complex relationships and latent features within the dataset. However, the combination method of Transformer encoding module and GCN does not outperform the evaluation metrics of MOSEGCN using both supervised loss and unsupervised loss utilizing unlabeled node information when only using supervised loss.This underscores the prowess of the SEGCN model within the MOSEGCN framework, effectively tapping into insights from unlabeled nodes to provide invaluable support during the model learning process. The symbiotic relationship between the Transformer encoding module and SEGCN not only highlights their collective strength but also opens up new horizons for pioneering advancements in the prediction and classification of intricate disease.

**Table 2:** Classification Results on the ROSMAP Dataset

Method	ACC	AUC	F1	Precision	Recall
RF	0.754	0.755	0.759	0.774	0.745
KNN	0.651	0.649	0.673	0.655	0.691
Lasso	0.755	0.751	0.783	0.723	**0.854**
XGBoost	0.764	0.763	0.775	0.768	0.782
MoGCN	0.774	0.773	0.784	0.791	0.790
MOGONET	0.800	**0.876**	0.801	0.832	0.775
Transformer+GCN	0.802	0.803	0.804	0.827	0.782
S3VM	0.774	0.775	0.772	0.809	0.739
SEGCN	0.792	0.794	0.792	0.824	0.764
MOSEGCN	**0.830**	0.832	**0.827**	**0.878**	0.782

**Table 3:** Classification Results on the BRCA Dataset

Method	ACC	F1_weighted	F1_macro	Precision	Recall
RF	0.768	0.756	0.697	0.731	0.675
KNN	0.783	0.777	0.732	0.801	0.692
Lasso	0.772	0.752	0.709	0.792	0.672
XGBoost	0.791	0.786	0.730	0.775	0.700
MoGCN	0.837	0.834	0.798	0.842	0.770
MOGONET	0.806	0.774	0.697	0.758	0.691
Transformer+GCN	0.840	0.834	0.784	0.836	0.755
S3VM	0.819	0.817	0.778	0.829	0.761
SEGCN	0.840	0.839	0.798	0.844	0.775
MOSEGCN	**0.867**	**0.868**	**0.811**	**0.874**	**0.797**

**Fig. 3:**
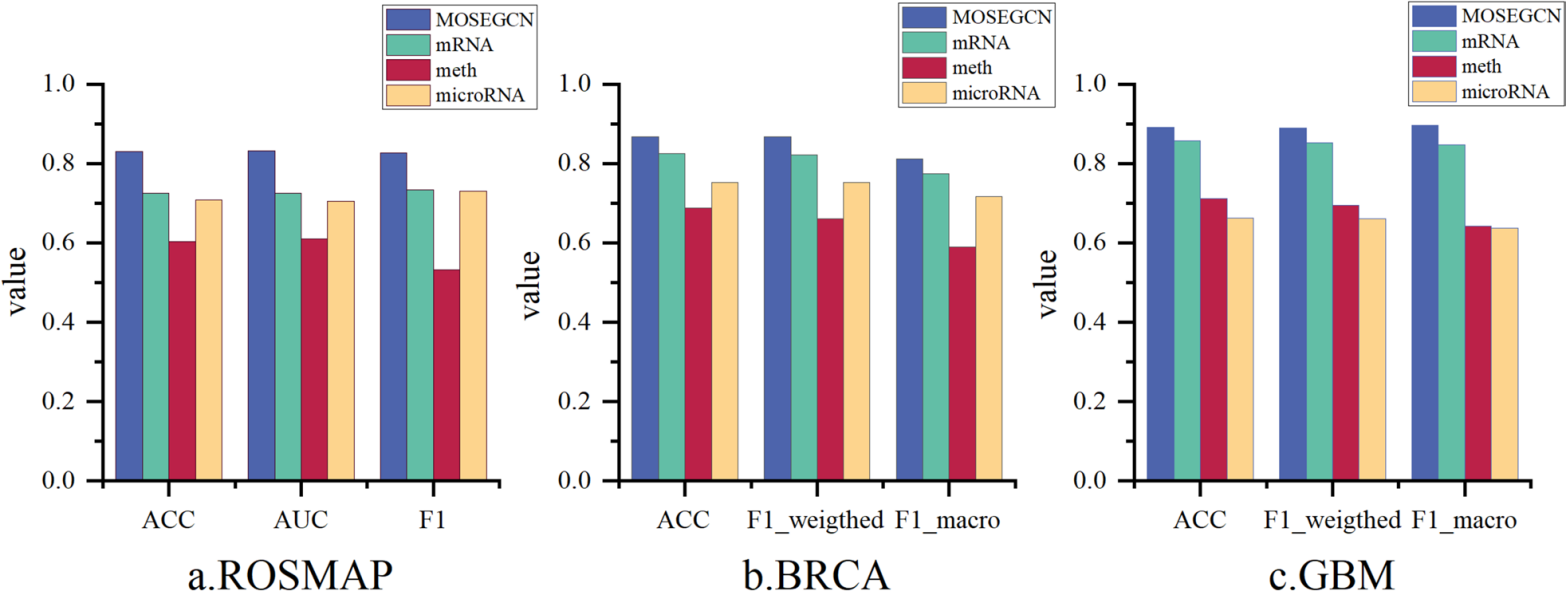
Comparison of Multi-Omic Data and Single-Omic Data Classification Results Using the MOSEGCN Model

MOSEGCN integrates three different types of omics data, and to demonstrate that MOSEGCN's classification performance surpasses that of single omics datasets, this experiment compares the classification results between single omics data and multi-omics data using MOSEGCN. As illustrated in Fig. 3, the results indicate that simultaneously processing all three omics data types yields the best classification results. This method of integrating multi-omics datasets considers information from multiple perspectives and levels, thereby enhancing the accuracy of classification predictions.

### Validation of MOSEGCN on the GBM dataset

To ascertain the generalizability of MOSEGCN, this experiment applied MOSEGCN to the GBM dataset, which encompasses four major subtypes: Classical, Mesenchymal, Proneural, and Neural [39]. The results are presented in (Table 4). Table 4 reveals that the proposed MOSEGCN model performs exceptionally well on the GBM dataset, achieving an accuracy of 89.2%, a weighted F1 score of 89.0%, and a macro F1 score of 89.7%. This performance surpasses all other comparative methods. These outcomes underscore the broad potential applicability of MOSEGCN for complex disease classification based on multi-omics data.

**Table 4:** Classification Results on the GBM Dataset

Method	ACC	F1_weighted	F1_macro	Precision	Recall
RF	0.807	0.804	0.800	0.840	0.790
KNN	0.757	0.755	0.754	0.789	0.774
Lasso	0.783	0.784	0.787	0.795	0.782
XGBoost	0.783	0.782	0.771	0.793	0.764
MoGCN	0.840	0.843	0.834	0.841	0.842
MOGONET	0.831	0.833	0.821	0.820	0.824
Transformer+GCN	0.855	0.859	0.853	0.868	0.867
S3VM	0.843	0.839	0.830	0.869	0.818
SEGCN	0.867	0.865	0.857	0.892	0.836
MOSEGCN	**0.892**	**0.890**	**0.897**	**0.905**	**0.884**

### Identification of significant biomarkers

Sensitivity analysis is a method employed to understand how neural network models respond to variations in input data. Through sensitivity analysis, one can ascertain the contribution of input features to output predictions and discern which input features exert the most significant influence on the model's predictive outcomes [40, 41]. The importance of a node can be determined by its feature's standard deviation (variable sensitivity) and its contribution to the network, referred to as weight sensitivity [42]. In the teacher model, this experiment employed sensitivity analysis for feature extraction. To achieve a stable feature extraction for the teacher model during training, the standard deviation ${\sigma }_{i}$ of each input node i's corresponding feature in each omics was calculated along with its connection weight *W*_ij_ in the network. Every 400 epochs, the top 30 markers were extracted, and the extracted features were consolidated. Table 5 enumerates the biomarkers associated with the classification of BRCA and ROSMAP datasets.

**Table 5:** Identified Important Biomarkers

Dataset		Omics type	important biomarkers
BRCA		mRNA expression	LIN28B|389421,TFF1|7031,CYP2B7P1|1556,FABP7|2173,TLX3|30012,SOX10|6663,ANKRD30A|91074,KRT6B|3854,CA9|768,CXorf61|203413,AGR3|155465,MIA|8190,GABRP|2568,GP2|2813,C1orf64|149563,SBSN|374897,KLK7|5650,PTPRZ1|5803,SFRP1|6422,KLK6|5653,ABCC11|85320,KLK8|11202,MSLN|10232,ZBTB16|7704,A2ML1|144568,TUBA3E|112714,SLC6A14|11254,C2orf40|84417,KRT16|3868,VGLL1|51442,C6orf218|221718,ZIC1|7545,CA6|765,HORMAD1|84072,TRIML2|205860,UPF0639|400224,TRIM15|89870,ISL2|64843,MAPK4|5596,ART3|419,RAET1L|154064,PSAPL1|768239
		DNA methylation	MIR124-2,SAMSN1,OR1J4,MIR563,FLJ41856,ZSWIM2,TAS2R13,LOC100130331,C5orf39,LOC145837,SLC5A12,SIRPD,MEP1A,POU4F1,FCGR2B,MIR100,SERPINB12,ARHGAP28,SCGB3A1,POU3F3,BLID,OR4N5,OR11G2,SLC22A2,DOK5,ZP4,CRISP2,LOC285692,KCNJ16,C14orf72,PSAT1,MT1DP,MIR365-1,TNFSF13B,INA,OR11H6,TAL2,DEFB118,S100A7,TMEFF1
		microRNA expression	hsa-mir-934,hsa-mir-449a,hsa-mir-577,hsa-mir-135b,hsa-mir-184,hsa-mir-190b,hsa-mir-187,hsa-mir-1269,hsa-mir-449b,hsa-mir-2115,hsa-mir-519a-1,hsa-mir-105-2,hsa-mir-105-1,hsa-mir-767,hsa-mir-205,hsa-mir-9-3,hsa-mir-375,hsa-mir-224,hsa-mir-210,hsa-mir-1251,hsa-mir-196a-1,hsa-mir-9-2,hsa-mir-486,hsa-mir-516a-2,hsa-mir-206,hsa-mir-196a-2,hsa-mir-4326,hsa-mir-135a-1,hsa-mir-452,hsa-mir-522,hsa-mir-137,hsa-mir-1304,hsa-mir-935,hsa-mir-937,hsa-mir-374c
ROSMAP	mRNA expression		FRMPD2P1 ,LINC01007,CTB-171A8.1,TAC3,S100A4,LINC00507,SLC5A11,RP11-552D4.1,LINC00499,RP11-298D21.1,DDIT4,BX255923.3,PHYHD1,APLN,ANLN ,RBP4,TGFBR3L,HSPA2,TF ,PNMA5,CXCR4,GAREML,CTD-2380F24.1,SCN3B,FAM65C,RP11-321E2.3 ,TRIP10,KCNJ10,RP11-416I2.1,UGT8
	DNA methylation		LDHC,SLC44A2,TRIP10 ,EMC4,CCL3,ENG,SLC44A2,ATG10,AGMAT,CCDC8,LRRC39,CMTM5,IRF7,ACSM5,LECT1,GFM1,HCAR1,EML2,TRAPPC12,SRRM2-AS1,HRC ,AHSP,C10orf11,EFS,XAF1,ECEL1,FBXL22,ARHGEF4,PTGER1,CDH1
	microRNA expression		hsa-miR-1246,hsa-miR-1299,hsa-miR-200a,ebv-miR-BART8,hsa-miR-520e,hsa-miR-1275,hsv1-miR-H8,hsa-miR-2117,hsa-miR-199a-5p,hsa-miR-330-3p,hsa-miR-1260,hsa-miR-744,hsa-miR-891b,hsa-miR-1308,hsa-miR-522,hsv1-miR-H3,hsa-miR-2114,hsa-miR-133b,hsa-miR-27a,hsa-miR-509-3p,mcv-miR-M1-5p,hsa-miR-153,hsv1-miR-H1,hsa-miR-208a,hsa-miR-1248,hsa-miR-639,hsa-miR-518e,hsa-miR-194,hsa-miR-199b-5p,hsa-miR-381

According to information from the KEGG database, we have discovered that in breast cancer, olfactory receptors such as OR11H6, OR1J4, OR4N5, and OR11G2O are associated with the olfactory transduction pathway. Olfactory receptors are not only expressed in the nasal cavity but also widely distributed throughout the body, playing significant physiological roles [43]. This finding suggests that these sensory receptors may serve as novel, yet insufficiently studied targets in the development and progression of breast cancer. The Estrogen Signaling Pathway plays a crucial role in BRCA1 [44], with KRT16 and TFF1 being part of this pathway. Their expressions influence the biological characteristics of BRCA. Enhanced KRT16 expression is significantly correlated with lower overall survival in metastatic breast cancer patients [45], while TFF1 is closely associated with bone metastasis in estrogen receptor (ER) + breast cancer [46]. PSAT1, CA6 and CA9 are part of the Metabolic Pathways [47] and affect the growth and migration of breast cancer cells [48–51] MIR100 [52, 53] and MIR124-2 [54, 55] are two MicroRNAs within the same signaling pathway that induce apoptosis and cell cycle arrest in breast cancer cells through multiple genes. In terms of microRNA, hsa-mir-135b has been identified as a target for treating AGR2-expressing breast cancer with doxorubicin resistance [56]. Gong et al. [57] formulated a prognostic risk feature model for predicting the prognosis of breast cancer patients. The results demonstrate a significant correlation between the expression levels of hsa-miR-190b and both unfavorable and favorable prognoses. In Alzheimer's disease, the Calcium Signaling Pathway is one of the major mechanisms. Disruption of calcium signaling may lead to synaptic defects and the accumulation of Aβ plaques and neurofibrillary tangles in AD [58, 59]. HRC, PTGER1 and CXCR4 are genes related to this pathway and have certain roles in the Calcium Signaling Pathway [60–62]. The Neuroactive Ligand-Receptor Interaction pathway may play a role in neuroactivity regulation and cognitive functions [63–65], with PTGER1, TAC3, and APLN being part of this pathway, influencing the nervous system in patients [66–68]. DDIT4 and ATG10 are involved in the Autophagy pathway, contributing to the clearance of cellular abnormalities by regulating the autophagic pathway [69–72]. Regarding microRNA, hsa-miR-199b-5p is a potential candidate biomarker for its role in the interaction between diabetes and Alzheimer's disease [73, 74] identified hsa-miR-133b as a potential biomarker for Alzheimer's disease (AD), playing a crucial role in constructing the ceRNA regulatory network associated with lncRNA. Hsa-miR-27a is likely a significant epigenetic biomarker in AD, participating in the regulation of the target gene SERPINA3, revealing its pivotal role in the disease's pathogenic mechanism [75].

## Discussion

Multi-omics data provides a diverse range of molecular-level insights into biological organisms. The comprehensive analysis of multi-omics data yields more thorough and accurate biological information. Furthermore, it uncovers novel biological insights and associations, fostering innovation in complex disease research. It propels data-driven biological studies and the advancement of personalized medicine. With the rapid advancement of omics technologies and healthcare standards, meticulously annotated omics datasets are on the rise. However, in the real world, the cost of extensively annotating data is often prohibitive, resulting in a small fraction of labeled data, leaving a substantial portion unlabeled. To address this challenge, this experiment introduces a deep learning-based semi-supervised multi-omics integration method for biomedical classification tasks. It effectively leverages both labeled and unlabeled data for improved classification of complex diseases. In this approach, we employed the Transformer encoding module for feature learning and integration. The Transformer network introduces a multi-head self-attention mechanism, allowing the model to establish connections between different positions. Moreover, this multi-head self-attention mechanism permits the model to consider the relevance of positions in the input data when generating representations for each position. This enhances the model's ability to learn hidden information between different omics data types, which is crucial for effectively integrating multi-omics features. Consequently, in this experiment, we concatenated various omics data to learn useful information at each position, encompassing both intra-modality and inter-modality internal feature information. For the final cancer classification, we employs SEGCN, which adeptly harnesses labeled and unlabeled data, enhancing the model's generalization capacity. The necessity of both key components, the Transformer encoding module and GCN, is verified through their combined use. The generalizability of MOSEGCN is validated on the GBM renal cell carcinoma dataset, where it demonstrates the ability to identify meaningful biomarkers within each omics data, elucidating certain disease-related information. MOSEGCN exhibits strong capabilities in integrating multi-omics data for cancer classification. However, it has limitations, as this study exclusively employed three distinct types of multi-omics data. Multi-omics data with more than three types and heterogeneous data, such as imaging omics, remain unverified. These areas represent future directions for further research.

## Conclusion

In conclusion, we introduces an innovative deep learning multi-omics integration model for the classification of complex diseases. Empirical evidence demonstrates the efficient utilization of the Transformer network to capture long-term dependencies in potential features within and across different modalities. Moreover, this experiment leverages the SEGCN module to thoroughly assimilate information from both labeled and unlabeled nodes, resulting in more precise classification outcomes. This integrated model is validated on three public datasets, outperforming state-of-the-art methods. Additionally, it identifies meaningful biomarkers within diverse omics data, further enhancing our understanding of disease mechanisms. In the future, we will explore different modalities and multi-omics data integration techniques to further enhance the performance of complex disease classification tasks.

## Data Availability

The BRCA and ROSMAP datasets analyzed in this study were obtained from Wang et al. [17]. The GBM dataset was downloaded from the provided link: http://acgt.cs.tau.ac.il/multi_omic_benchmark/download.html.
